# Genetic Spectrum and Variability in Chinese Patients with Amyotrophic Lateral Sclerosis

**DOI:** 10.14336/AD.2019.0215

**Published:** 2019-12-01

**Authors:** Zhi-Jun Liu, Hui-Xia Lin, Qiao Wei, Qi-Jie Zhang, Cong-Xin Chen, Qing-Qing Tao, Gong-Lu Liu, Wang Ni, Aaron D Gitler, Hong-Fu Li, Zhi-Ying Wu

**Affiliations:** ^1^Department of Neurology and Research Center of Neurology in Second Affiliated Hospital, and Key Laboratory of Medical Neurobiology of Zhejiang Province, Zhejiang University School of Medicine, Hangzhou, China.; ^2^Department of Neurology and Institute of Neurology, First Affiliated Hospital, Fujian Medical University, Fuzhou, China.; ^3^Department of Genetics, Stanford University School of Medicine, Stanford, CA, USA.

**Keywords:** amyotrophic lateral sclerosis, mutation, targeted next-generation sequencing, Chinese ancestry, genetic spectrum

## Abstract

Amyotrophic lateral sclerosis (ALS) is a progressive, fatal neurodegenerative disease characterized by selective impairment of upper and lower motor neurons. We aimed to investigate the genetic spectrum and variability in Chinese patients with ALS. A total of 24 familial ALS (FALS) and 21 early-onset sporadic ALS (SALS) of Chinese ancestry were enrolled. Targeted next-generation sequencing (NGS) was performed in the probands, followed by verification by Sanger sequencing and co-segregation analysis. Clinical features of patients with pathogenic or likely pathogenic variants were present. The mutation frequency of ALS-related genes was then analyzed in Chinese population. In this cohort, 17 known mutations (9 *SOD1*, 5 *FUS*, 2 *TARDBP* and one *SETX*) were identified in 14 FALS and 6 early-onset SALS. Moreover, 7 novel variants (*SOD1* c.112G>C, *OPTN* c.811C>T, *ERBB4* c.965T>A, *DCTN1* c.1915C>T, *NEFH* c.2602G>A, *NEK1* c.3622G>A, and *TAF15* c.1535G>A) were identified. In southeastern Chinese FALS, the mutation frequency of *SOD1*, *FUS*, and *TARDBP* was 52.9%, 8.8%, 8.8% respectively. In early-onset SALS, *FUS* mutations were the most common (22.6%). In Chinese ALS cases, p.H47R is most frequent *SOD1* mutations, while p.R521 is most common *FUS* mutation and p.M337V is most common *TARDBP* mutation. Our results revealed that mutations in *SOD1, FUS* and *TARDBP* are the most common cause of Chinese FALS, while *FUS* mutations are the most common cause of early-onset SALS. The genetic spectrum is different between Chinese ALS and Caucasian ALS.

Amyotrophic lateral sclerosis (ALS) is a progressive, fatal neurodegenerative disease characterized by selective impairment of upper and lower motor neurons, with or without cognitive impairment [1]. It predominantly occurs in adulthood with irreversible course. Patients usually suffer from progressive muscle weakness and atrophy and die of respiratory failure 3-5 years after the onset. No medications can halt the progression of ALS, although Riluzole prolongs survival by several months [2]. Approximately, 5-10% of cases are familial ALS (FALS) with a Mendelian inheritance, while the remaining cases are sporadic ALS (SALS) [3]. The etiology of ALS has not been fully uncovered so far, but growing studies have revealed that genetic mutations played important roles in the pathogenesis of ALS, especially in juvenile-onset ALS [4]. In recent years, evolving technologies for gene mapping and sequencing have facilitated the identification of multiple ALS genes. To date, more than 25 genes have been implicated in ALS [5,6]. Among these genes, *C9ORF72*, *SOD1*, *FUS*, and *TARDBP* are the most common causative genes [4]. Although mutations in some ALS-linked genes have been observed worldwide, geographic variability has also been observed. For example, the *C9ORF72* hexanucleotide repeat expansion accounts for about 40% of FALS in North America and Europe [4]. while it is rare in the Chinese ALS population [7,8]. Such discrepancies might be attributed to differences in ethnicity and haplotype background.

In this study, we performed targeted next-generation sequencing (NGS) on a cohort of 24 FALS cases and 21 early-onset SALS cases from southeastern China. In addition, we integrated the previously reported ALS cases from our group and other Chinese groups to characterize the genetic spectrum of Chinese ALS patients. We summarize the mutation frequency and distribution of common ALS causative genes (*SOD1*, *FUS*, and *TARDBP*) in Chinese population.

## MATERIALS AND METHODS

### Participants

A total of 24 FALS probands and 21 early-onset sporadic ALS were recruited from July 2008 to April 2017 in Second Affiliated Hospital, Zhejiang University School of Medicine, First Affiliated Hospital, Fujian Medical University, and Huashan Hospital, Fudan University. Majority of patients were from southeastern China. Age at onset under 40 years old was defined as early-onset in this study. Of the 24 FALS cases, five cases were previously reported, but no responsible mutation was found that time [9,10]. The diagnosis of ALS was made by at least two senior neurologists. All patients fulfilled the El Escorial criteria for ALS [11]. In view of the fact that a few patients with a diagnosis of suspected ALS had predominant lower motor neuron (LMN) symptoms, we excluded the possibility of spinal muscular atrophy (SMA) and spinal-bulbar muscular atrophy (SBMA) by screening *SMN1* and *AR* gene in all patients. This study was approved by the ethics committee at each participating center. Written consents were obtained from all participants.

### Targeted sequencing

Genomic DNA was extracted from EDTA-treated peripheral blood using DNA Extraction Kit (Qiagen, Hilden, Germany). Genetic analysis of the hexanucleotide repeats in *C9ORF72* was completed as previously described [12]. A custom panel (Roche, Madison, USA) was designed to capture and sequence the 27 well-established ALS-related genes (Supplementary table 1). All the exons and flanking regions of each gene were covered by this panel. The targeted exomes of the prepared samples were captured by NimbleGen SeqCap EZ products (Roche, Madison, USA). The targeted libraries were indexed, polled and sequenced using Illumina Hiseq2500 system (Genergy Biotechnology Co. Ltd., Shanghai, China). Short reads were aligned to the human genome (UCSC hg19) using Burrows-Wheeler Aligner v0.7.15. The calling of variants, including single nucleotide variants (SNVs) and indels, were accomplished by Genome Analysis Toolkit v3.6. Sequencing depth and coverage analyses were performed using Genome Analysis Toolkit v3.6. The called variants were filtered by Hard filtering and Variant Quality Score Recalibration, and further annotated using ANNOVAR v2016Feb01.

**Table 1:** Demographic and clinical features of all ALS patients.

	FALS	SALS	Total
Number of subjects, n	24	21	45
M/F, n	13/11	13/8	26/19
M/F ratio	1.2	1.6	1.4
Age of onset, mean ± SD (y)			
M	34.5±13.1	30.4±13.5	32.4±13.2
F	47.1±14.1	31.3±8.1	40.4±14.2
Total	40.3±14.8	30.7±11.5	35.8±14.1
Site of onset, n			
Upper limbs	9	11	20
Lower limbs	13	9	22
Chest/abdomen	1	0	1
Bulbar	1	1	2

### Variant analysis

All nonsynonymous variants covered more than 20x and within the coding and splicing regions were selected and screened with dbSNP, 1000 Genomes Project (1000G), NHLBI Exome Sequencing Project (ESP6500), and Exome Aggregation Consortium (ExAC). Potential functional consequences of the nonsynonymous variants were evaluated by SIFT, Mutationtaster, and PolyPhen-2, and CADD. The variants known to be pathogenic to the disease in any database of NCBI ClinVar, HGMD (Human Gene Mutation Database), and ALSoD (Amyotrophic Lateral Sclerosis Online Genetics Database) were picked out for further analysis.

**Table 2:** The known pathogenic variants of ALS-related gene identified in this study.

Gene	Nucleotide change	Amino acid change	Index cases (family history)
*SOD1*	c.13G>T	p.A5S	1 (Yes)
*SOD1*	c.14C>T	p.A5V	1 (Yes)
*SOD1*	c.32G>T	p.G11V	1 (Yes)
*SOD1*	c.49G>T	p.G17C	1 (Yes)
*SOD1*	c.140A>G	p.H47R	2 (Yes)
*SOD1*	c.251A>G	p.D84G	1 (Yes)
*SOD1*	c.319C>G	p.L107V	1 (Yes)
*SOD1*	c.363T>G	p.H121Q	2 (Yes)
*SOD1*	c.449T>C	p.I150T	1 (Yes)
*FUS*	c.1483C>T	p.R495X	1 (Yes)
*FUS*	c.1528A>G	p.K510E	1 (No)
*FUS*	c.1561C>T	p.R521C	1 (No)
*FUS*	c.1562G>A	p.R521H	1 (Yes)
*FUS*	c.1574C>T	p.P525L	2 (No)
*TARDBP*	c.1009A>G	p.M337V	1 (Yes)
*TARDBP*	c.1069G>A	p.G357S	1 (No)
*SETX*	c.1504C>T	p.R502W	1 (No)

### Sanger sequencing

Sanger sequencing was performed as previously described to verify the variants identified by targeted NGS [13]. Briefly, forward and reverse PCR primers were designed to amplify the fragments covering the variant sites. PCR products were purified with shrimp alkaline phosphatase and exonuclease and then directly sequenced on an ABI 3500xL Dx Genetic Analyzer (Applied Biosystems, Foster City, USA). Co-segregation analysis was performed in the families with identified mutations.

## RESULTS

### Overview of demographics and clinical features

A total of 24 FALS probands (13 males; 11 females) and 21 early-onset SALS (13 males; 8 females) were included in this study. The demographic and clinical features were summarized in Table 1. All patients are of Chinese Han origin and the majority of patients were from southeastern China. Among these patients, the mean age at onset was 35.8±14.1 years (range from 7 to 65). Site of onset was upper limbs in 20 cases and lower limbs in 22 cases. Only two cases had bulbar onset and one case had abdomen onset.

### Targeted sequencing of ALS patient samples

Among the 45 subjects recruited to our study, we subjected each DNA sample to next generation sequencing (NGS). An average of 93.25% target regions were sequenced with at least 30x coverage, and 86.18% target regions with 50x coverage. The mean coverage of the target regions per sample was 179.73. After filtering, a total of 29 different variants including 17 known pathogenic variants and 7 novel variants were identified (Table 2 and 3). All these variants were verified by Sanger sequencing.

### Seventeen known pathogenic variants were identified

We assessed the *C9ORF72* hexanucleotide repeat expansion by repeat-primed PCR, resulting in negative findings in all samples, consistent with the paucity of *C9ORF72* mutation carriers in the Chinese ALS population [8]. A total of 17 known pathogenic variants, including 9 *SOD1* mutations (c.13G>T, c.14C>T, c.32G>T, c.49G>T, c.140A>G, c.251A>G, c.319C>G, c.363T>G and c.449T>C), 5 *FUS* mutations (c.1483C>T, c.1528A>G, c.1561C>T, c.1562G>A, and c.1574C>T), 2 *TARDBP* mutations (c.1009A>G and c.1069G>A), and one *SETX* mutation (c.1504C>T), were identified in 14 FALS cases and 6 early-onset sporadic cases (Table 2). In addition, we detected a previously reported *SETX* variant (c.4660T>G, p.C1554G) in a sporadic case. This variant was documented in a few studies with controversial pathogenicity [14-16]. The frequency of this variant is 0.0058 in 1000G, 0.0032 in ESP6500, and 0.0058 in ExAC. Co-segregation analysis revealed that this variant was also present in the patient’s unaffected mother and younger brother. Therefore, this variant seems unlikely to be pathogenic.

### Seven novel variants were identified in ALS-related genes

In addition, we identified 7 novel variants in 7 unrelated ALS cases, including 5 FALS and two early-onset SALS (Table 3). All these variants were absent or with low frequency in 1000G, ESP6500, and ExAC. Of these variants, one *SOD1* variants (c.112G>C) were identified in one FALS cases. The mutation was absent in 1000G, ESP6500, ExAC, and our 200 controls. In addition, it was predicted to be deleterious by SIFT, PolyPhen-2, Mutation Taster, and CADD. Then, another *SOD1* mutation (c.112G>A) has been reported to be pathogenic (http://www.hgmd.cf.ac.uk/). According to ACMG standards, this variant was classified as pathogenic. Six variants (*OPTN* c.811C>T, *ERBB4* c.965T>A, *DCTN1* c.1915C>T, *NEFH* c.2602G>A, *NEK1* c.3622G>A, and *TAF15* c.1535G>A) of uncertain significance were identified in four FALS patients and two early-onset SALS patients. In addition, a novel variant (c.-34C>A) in a noncoding region of *FUS* was identified in a sporadic case.

Altogether, 17 known pathogenic variants and one novel pathogenic variant was detected in 15 FALS cases and 6 SALS cases. In addition, 6 variants of uncertain significance were detected in four FALS cases and two early-onset SALS cases. In total, 19 of 24 (79.2%) FALS and 8 of 21 (38.1%) early-onset SALS cases were identified to carry variants of ALS-related genes, while no variant was detected in the remaining cases.

**Table 3:** The novel variants of ALS-related genes identified in this study.

Gene	Mutation	1000G	ESP6500	ExAC	SIFT	Mutation Taster	Polyphen-2	CADD	ACMG
*SOD1*	c.112G>C (p.G38R)	0	0	0	Damaging	Disease causing	Probably damaging	Damaging	P(PS1+PM1+PM2+PP2+PP3)
*OPTN*	c.811C>T (p.R271C)	0	0	1.65E-05	Tolerable	Polymorphism	Benign	Damaging	VUS (PM2)
*ERBB4*	c.965T>A (p.M322K)	0	0	0	Tolerable	Disease causing	Benign	Tolerable	VUS (PM1+PM2+PP2)
*DCTN1*	c.1915C>T (p.R639W)	0	0	4.99E-05	Damaging	Disease causing	Probably damaging	Damaging	VUS (PM1+PM2+PP3)
*NEFH*	c.2602G>A (p.E868K)	0	0	0	Tolerable	Disease causing	Probably damaging	Tolerable	VUS (PM2+PP3+BP1)
*NEK1*	c.3622G>A (p.D1208N)	0	0	1.06E-04	Damaging	Disease causing	Probably damaging	Damaging	VUS (PM2)
*TAF15*	c.1535G>A (p.R512Q)	0	7.7E-05	9.15E-05	Damaging	Disease causing	Probably damaging	Damaging	VUS

Abbreviations: 1000G= 1000 Genomes Project; ESP6500= NHLBI exome sequencing project; ExAC= Exome Aggregation Consortium.

### Clinical features of the patients carrying pathogenic variants

The clinical features of the patients carrying pathogenic variants or likely pathogenic variants are summarized in Table 4. The average age at onset is 37.81 ± 15.20 years, ranging from 13 to 65 years. Among the 21 patients carrying mutations, 19 cases presented with limb-onset symptoms, one case exhibited bulbar onset, and the other one initially developed weakness of abdominal muscles. Only one patient carrying a *FUS* p.K510E mutation exhibited cognitive decline four months after disease onset.

In the current study, 50% (12/24) of FALS cases carried *SOD1* mutations, including 9 known pathogenic variants (p.A5S, p.A5V, p.G11V, p.G17C, p.H47R, p.N84G, p.L107V, p.H121Q and p.I150T) and one novel pathogenic variant (c.112G>C, p.G38R). The average age of onset of *SOD1* mutation carriers was 42.75?±?13.44 years. The site of onset in *SOD1* mutation carriers was in the lower limbs (75%, n=9), upper limbs (16.7%, n=2), and abdominal muscle (8.3%, n=1). These patients typically presented with dominant lower motor neuron signs. Interestingly, slow progression was significantly observed in the patients carrying *SOD1* p.G38R, p.H47R, or p.D84G mutations.

Additionally, we found 5 known *FUS* mutations (p.R495X, p.K510E, p.R521C, p.R521H, and p.P525L) in two FALS cases and four SALS cases. The average age at onset in patients with *FUS* mutations was 25.67?±?7.61 years; significantly lower than that in patients with *SOD1* mutations. The patients carrying p.R521C or p.R521H mutation manifested predominant lower motor neuron signs. Interestingly, neck and proximal muscle weakness which usually occurs in p.R521C mutation carriers was also observed in our patient with p.R521H mutation. In line with previous reports [17-19], the patient with the p.R495X mutation showed an early-onset and rapid disease progression.

Moreover, we detected two known *TARDBP* mutations, p.M337V and p.G357S. The patient with p.M337V mutation exhibited onset of bulbar symptoms, while the patient with p.G357S had spine onset. Interestingly, we identified a known *SETX* variant (p.R502W) in a SALS patient, who predominantly presented muscle weakness and atrophy of proximal lower limbs and exhibited a slowly progressive disease course without any evidence of upper motor neuron signs.

**Table 4:** The clinical features of the patients carrying pathogenic variants or likely pathogenic variants.

Patients	Mutation	Family history	Gender	Age at onset (y)	Site of onset	Disease duration (m)	Age of death (y)	Predominant features
1	*SOD1*: c.13G>T	Yes	M	29	LL	>4	alive	LMN dominance
2	*SOD1*: c.14C>T	Yes	F	60	UL	15	61	Classical ALS
3	*SOD1*: c.32G>T	Yes	F	25	LL	>10	alive	Classical ALS
4	*SOD1*: c.49G>T	Yes	M	49	Abdomen	38	52	LMN dominance
5	*SOD1*: c.112G>C	Yes	M	26	LL	>120	alive	LMN dominance; slow progression
6	*SOD1*: c.140A>G	Yes	F	60	UL	>180	alive	LMN; very slow progression
7	*SOD1*: c.140A>G	Yes	M	53	LL	>21	alive	LMN dominance
8	*SOD1*: c.251A>G	Yes	M	32	LL	>29	alive	LMN dominance; slow progression
9	*SOD1*: c.319C>G	Yes	M	41	LL	>8	alive	Classical ALS
10	*SOD1*: c.363T>G	Yes	F	42	LL	51	46	LMN dominance
11	*SOD1*: c.363T>G	Yes	F	60	LL	27	62	Classical ALS
12	*SOD1*: c.449T>C	Yes	F	36	LL	12	37	Classical ALS
13	*FUS*: c.1483C>T	Yes	M	25	LL	9	26	Fast progression
14	*FUS*: c.1528A>G	No	M	26	UL	>14	alive	LMN dominance; cognitive decline
15	*FUS*: c.1561C>T	No	M	30	LL	41	33	LMN dominance
16	*FUS*: c.1562G>A	Yes	F	36	UL	>16	alive	Classical ALS; dropped head syndrome
17	*FUS*: c.1574C>T	No	F	13	LL	>40	alive	Classical ALS
18	*FUS*: c.1574C>T	No	M	24	UL	>27	alive	Classical ALS
19	*TARDBP*: c.1009A>G	Yes	M	44	Bulbar	>156	alive	LMN dominance; slow progression
20	*TARDBP*: c.1069G>A	No	M	65	UL	>24	alive	Classical ALS
21	*SETX*: c.1504C>T	No	M	18	LL	>84	alive	LMN dominance; slow progression

Abbreviations: M=male; F=female; LL= lower limb; UL=upper limb; LMN=lower motor neuron; ALS=amyotrophic lateral sclerosis.

### Genetic spectrum in our ALS patients from southeastern China

**Figure 1.**
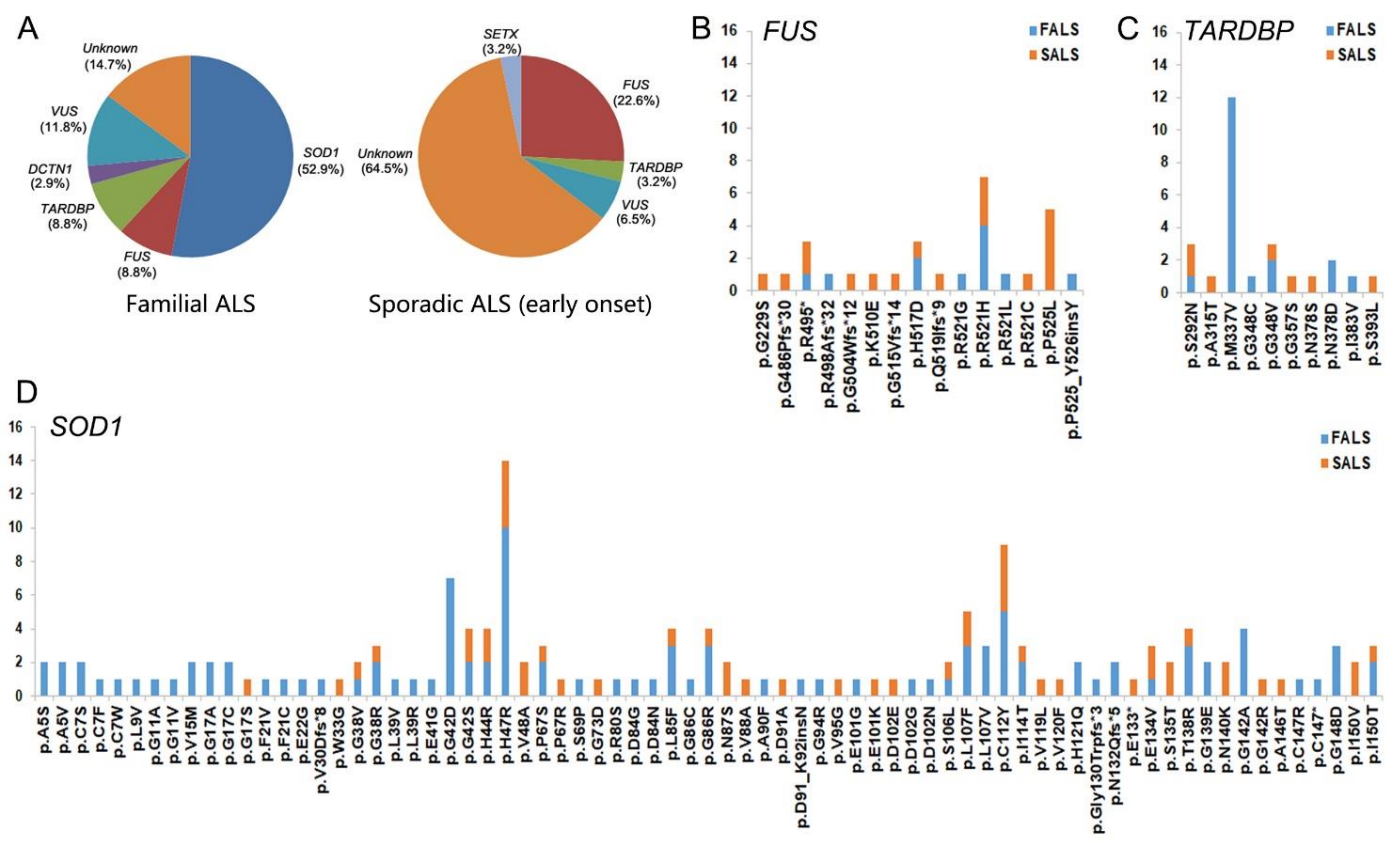
Mutation spectrum of ALS-linked genes in our ALS cohort and Chinese ALS patients. (A) In our ALS cohort, the mutation frequency of *SOD1*, *FUS*, *TARDBP*, and *DCTN1* in FALS was 52.9%, 8.8%, 8.8%, and 2.9%, respectively. *FUS* mutations were the most common cause of early-onset SALS (25.8%), while 61.3% of the cases were not identified pathogenic mutations. (B) The mutation frequency of *FUS* in Chinese ALS cases. (C) The mutation frequency of *TARDBP* in Chinese ALS cases. (D) The mutation frequency of *SOD1* in Chinese ALS cases.

We previously reported 10 unrelated FALS patients with mutations identified in *SOD1* (4 mutations/6 patients), *TARDBP* (2/2), *DCTN1* (1/1), and *FUS* (1/1) [9,10,20,21]. As shown in Figure 1A, when integrating these results into the current study, we found 85.3% (29/34) of FALS carried mutation. *SOD1* mutations were the most common (18/34, 52.9%) in our FALS cases, followed by *FUS* (3/34, 8.8%) and *TARDBP* (3/34, 8.8%) mutations. Despite being an uncommon cause, *DCTN1* mutations were identified in 2.9% (1/34) of FALS patients. However, 11.8% (4/34) of FALS cases carried a variant of unknown significance and 14.7% (5/34) of FALS patients were not identified potential pathogenic variants. In addition, we identified three *FUS* mutations in four SALS cases and one *TARDBP* mutation in one SALS case. Integrating our previously reported three *FUS* mutations in 3 of 10 juvenile-onset SALS cases [20], we found the frequency for *FUS* mutation and *TARDBP* mutation in early-onset SALS were 22.6% (7/31) and 3.2% (1/31), respectively. Also, one *SETX* mutation was detected in one SALS patient and two variants of uncertain significance were detected in two SALS cases. In total, 35.5% (11/31) of early-onset SALS patients were identified to carry potential pathogenic variants whereas the remaining 64.5% (20/31) of SALS patients were not.

### Mutation frequency of common ALS genes in Chinese population

To analyze mutation frequency and distribution of common ALS causative genes (*SOD1*, *FUS*, and *TARDBP*) in Chinese population, we reviewed the published genetic reports on Chinese ALS patients. In total, 36 additional studies reporting mutations of *SOD1*, *FUS*, and *TARDBP* in Chinese ALS patients were analyzed. As shown in Supplementary table 2, a total of 73 different *SOD1* mutations were reported in Chinese ALS patients (Fig. 1B). Among these mutations, p.H47R was detected in 14 unrelated patients, representing the most frequent *SOD1* mutation in Chinese ALS patients. In addition, *SOD1* mutations p.G42D, p.L107F, and p.C112Y are prevalent. The most common *SOD1* mutations in North America (p.A5V) and worldwide (p.D91A) were rare in Chinese ALS patients. A total of 15 *FUS* mutations including 6 indels, 8 missense mutations and one nonsense mutation were reported in Chinese ALS patients (Fig. 1C). The p.R521 mutation (including p.R521G, p.R521H, p.R521L and p.R521C) was found in 9 unrelated ALS patients, representing the most frequent *FUS* mutation in Chinese ALS patients. In addition, 10 *TARDBP* missense mutations were reported in Chinese ALS patients (Fig. 1D), all of which were located in exon 6. Of these *TARDBP* mutations, p.M337V seems to be a high frequency mutation in Chinese ALS patients. In early-onset SALS, 5 cases with *SOD1* mutations, 16 cases with *FUS* mutations, and one case with *TARDBP* mutation had been described.

## DISCUSSION

In this study, we performed targeted NGS and Sanger sequencing in a Chinese cohort of 24 FALS cases and 21 early-onset SALS cases. In total, we identified 17 known pathogenic variants and a novel *SOD1* pathogenic variants (c.112G>C) in 15 FALS cases and 6 early-onset SALS cases. In addition, 6 variants of uncertain significance were identified in four FALS cases and two SALS cases. The pathogenicity of these 6 novel variants remains to be determined. When integrating our previously reported ALS cases with mutation [9,10,20,21], we found that 85.3% (29/34) of FALS carried mutations of ALS-linked genes. However, we failed to detect pathogenic mutations in the majority (64.5%, 20/31) of early-onset SALS cases. Among these mutation carriers, *SOD1* mutation was the most common cause of FALS, which is consistent with previous studies [22,23,24,25]. *FUS* mutation accounted for the most frequent in the early-onset SALS cases. Previous studies of Chinese SALS which included patients with a wide range of age of onset indicated that *FUS* mutations are also the most frequent cause of SALS, but the frequency of *FUS* mutation in SALS is lower than that in early-onset SALS (25.8%), ranging from 1.7% to 1.9% [23,24]. However, none *FUS* mutations was identified in the cohorts of SALS from central-southern and northern China [25, 26]. Of note, no *C9ORF72* repeat expansion were identified in our cohort, further supporting the finding that *C9ORF72* mutation is rare in the Chinese population [8].

As the first causative gene for ALS, SOD1 mutations are very common in both FALS (20%) and SALS (1~2%) [27]. To date, more than 185 *SOD1* mutations have been described worldwide and p.D91A (previously described as p.D90A) is the most common one [28]. However, this mutation was reported in only one Chinese ALS case [23]. Another mutation p.A5V (previously described as p.A4V), which accounts for up to 50% of *SOD1* mutations in North America, is rarely described in Chinese ALS cases [29]. Moreover, it is reported that the p.A5V mutation carrier did not share the common founder effect observed in North America [29]. In contrast, p.H47R was the most common *SOD1* mutation in Chinese ALS cases, which is similar to that in Japan [30]. In addition, p.G42D, p.L107F, and p.C112Y, were more frequent than other mutations. These results imply that the mutation spectrum of *SOD1* was different between Chinese ALS and Caucasian ALS. In regard to the clinical features, cognitive impairment and bulbar onset was not common in Chinese patients with *SOD1* mutations, which is consistent with previous reports [30]. However, phenotypic heterogeneity was observed not only in cases with different *SOD1* mutations, but also in familial members with the same *SOD1* mutation.

Compared to the high frequency of *SOD1* mutations in Chinese FALS cases, *FUS* mutations were not common. However, *FUS* remains the most frequent genetic determinant of early-onset SALS. Previous study in China demonstrated that mutations in the *FUS* are the most frequent genetic cause in juvenile SALS (age of onset < 25 years) of Chinese origin [31]. This is consistent with our results. The frequency of *FUS* mutations in early-onset SALS is similar between Chinese and Caucasian population [32]. Of note, over half of *FUS* mutations in Chinese cases were frameshift mutations. Age at onset is young in most of the cases, and most of *FUS* mutation carriers manifested predominant lower motor neuron symptoms and a rapid disease progression. In addition, 9 mutations located in exon 6 of *TARDBP* were reported in ALS patients of Chinese ancestry and p.M337V seems to be the most common one, implying *TARDBP* is one of the major causative genes in Chinese ALS cases.

In summary, we investigated the genetic mutations in a Chinese cohort with ALS and characterize the genetic spectrum of Chinese ALS. Our results revealed that *SOD1* mutations are the most common cause of Chinese FALS, followed by *TARDBP, FUS* and *DCTN1*. *FUS* mutations remained the most frequent genetic determinant of Chinese early-onset SALS. In the future, it will be interesting to explore the basis for variability in mutation frequencies between Chinese ALS and Caucasian ALS.

## Supplementary Materials

The Supplemenantry data can be found online at: www.aginganddisease.org/EN/10.14336/AD.2019.0215.
